# Post-Pandemic Virtual Teaching Self-Efficacy: Insights from Medical Educators at Shiraz University of Medical Sciences, Iran

**DOI:** 10.34172/mejdd.2025.431

**Published:** 2025-07-30

**Authors:** Ali Reza Rezvani, Nahid Zarifsanaiey, Parviz Shahmirzalou, Manoosh Mehrabi, Leila Rahmati, Ali Reza Safarpour

**Affiliations:** ^1^Hematology Research Center, Shiraz University of Medical Sciences, Shiraz, Iran; ^2^Department of e-Learning in Medical Sciences, Virtual School, Comprehensive Center of Excellence for e-Learning in Medical Sciences, Shiraz University of Medical Sciences, Shiraz, Iran; ^3^Public Health Department, Khoy University of Medical Sciences, Khoy, Iran; ^4^Gastroenterohepatology Research Center, Shiraz University of Medical Sciences, Shiraz, Iran

**Keywords:** Self-efficacy, Virtual learning, Medical education

## Abstract

**Background:**

The COVID-19 pandemic has highlighted the importance of teacher self-efficacy in online instruction, with virtual teaching models associated with lower efficacy scores compared to in-person formats. This study assessed the self-efficacy of faculty members at Shiraz University of Medical Sciences (SUMS) in online teaching, with a focus on the impact of demographic factors and specialty areas on virtual teaching efficacy post-COVID-19.

**Methods:**

This cross-sectional analytic study was conducted between July 2023 and August 2024, on 203 clinical faculty members from Shiraz University of Medical Sciences. Participants included professors with at least one year of clinical experience and who were actively teaching in one of the university’s hospitals. The Teachers’ Sense of Self-Efficacy Scale (TSES), developed by Tanchan-Moran and Hoy, was used to assess self-efficacy. The instrument was translated from English to Farsi using forward-backward translation methods. Internal consistency and validity were confirmed. Descriptive statistics, one-way ANOVA, t-tests, and chi-square tests were used to analyze the data.

**Results:**

The average (SD) work experience of participants was 15.11 (8.89) years, with most being male, married, and working in the medical field. The internal consistency of TSES was excellent (Cronbach’s alpha is 0.94). The average (SD) self-efficacy score of participants was 56.17 (14.62), with a minimum score of 22 and a maximum score of 110. Additionally, no significant regression relationships were found between demographic factors (sex, work experience, field of activity, and marital status) and the self-efficacy score. Approximately 70% of the faculty members reported a moderate to high self-efficacy in virtual teaching.

**Conclusion:**

This study provides valuable insights into the self-efficacy of SUMS faculty members in online education following the COVID-19 pandemic. While the study recommends that overall self-efficacy levels are moderately high, there is an ongoing need for continued investment in faculty development programs and support to confirm effective online teaching practices and address the evolving needs of medical education.

## Introduction

 The COVID-19 pandemic has significantly altered the field of higher education, compelling organizations worldwide to adopt online learning methods on an unprecedented scale.^1^ This rapid change created many kinds of problems, especially for faculty members who used face-to-face teaching methods. The indispensability of online learning also encouraged studies on the willingness and effectiveness of educators in adjusting to this new educational model.^2^ The COVID-19 pandemic has highlighted the importance of teacher self-efficacy in online instruction, with virtual teaching models associated with lower efficacy scores compared to hybrid or in-person formats.^3^

 A crucial factor impacting the effectiveness of online learning is faculty self-efficacy, which refers to the belief in one’s own capacity to effectively teach in an online situation. In affecting instructional approaches, motivation, and ultimately, student learning results, self-efficacy plays a crucial role.^4^ In the setting of online learning, faculty self-efficacy is largely important as it can impact their capability to: design engaging and effective online courses, adjust teaching strategies to the online environment, provide effective support to online learners, and encourage online student collaborations.

 Despite the critical role of faculty self-efficacy in online teaching, the literature suggests that many faculty members struggle with confidence and competence in this new environment.^5^ This is mainly true for those who were thrust into online teaching without satisfactory preparation or support.^6^ The pandemic’s rapid shift to online learning highlighted the necessity for a better insight into faculty self-efficacy and the aspects that impacted it, particularly in the setting of returning to in-person teaching procedures after the initial crisis.^7^

 This study is grounded in Bandura’s Social Cognitive Theory (SCT), which posits that an individual’s confidence in their own abilities (self-efficacy) plays a crucial role in determining their activities, motivations, and ultimately, their successes. The SCT provides a theoretical foundation for understanding how faculty self-efficacy in online teaching influences their educational practices, their interactions with students, and their overall effectiveness in the online learning environment.

 This study assessed the self-efficacy of faculty members at Shiraz University of Medical Sciences (SUMS) in online teaching, with a focus on the impact of demographic factors and specialty areas on virtual teaching efficacy following the COVID-19 pandemic.

## Materials and Methods

 This cross-sectional analytical study, conducted between July 2023 and August 2024, involved 203 clinical faculty members affiliated with Shiraz University of Medical Sciences, who participated and completed the questionnaire.

 All faculty members with at least one year of work experience and clinical expertise, working in one of the hospitals of Shiraz University of Medical Sciences, with no age restriction, were eligible. Those who did not want to participate in this study or worked in non-clinical groups were excluded. The Teachers’ Sense of Self-Efficacy Scale (TSES) questionnaire was used in this study.^4^ This questionnaire measured three dimensions of self-efficacy among the participants, including: 1) planning for teaching, class activities, and evaluation, 2) classroom presentation and management, and 3) teaching works. The questionnaire, containing 22 questions based on a 5-point Likert scale, ranging from “no effect” to “very effective,” asked participants for their opinions. The internal consistency and validity of the instrument were checked during the study. The original English version has been translated into Persian, including both forward and backward translations. We conducted a pilot study with the translated version of the questionnaire on 20 SUMS faculty participants to assess its clarity, comprehensibility, and cultural appropriateness. At this stage, the pilot study results confirmed the excellent validity and reliability (Cronbach’s alpha = 0.94) of the Persian version of the TSES. To calculate the validity, 25 items in the translated version were sent to six expert professors in the field of education. Based on the content validity index (CVI) formula, CVI was calculated. The items with CVI below 0.8 were either removed or revised, and finally, 22 items of the questionnaire were approved. The pilot study, conducted during the main study, is a well-established standard method for preliminary validation of translated instruments, as evidenced by similar studies in other countries.^8-10^ The sample size was calculated using G-power software. Considering a power of 90%, a type 1 error of 5%, and a medium effect size, the sample size was calculated to be 180 people. According to the application of a 10% loss, this was increased to 198 participants.

 We sent the questionnaire via email to 500 faculty members, and 203 people answered our email and agreed to attend the study. Before the study, the respondents were informed about the study’s aim, their ability to withdraw at any time, and the privacy of their answers. Participation was voluntary, and written informed consent was obtained. The ethics committee code is: IR.SUMS.REC.1402.470

###  Statistical Analysis

 Statistical analysis includes five parts. First, the demographic variables were described in terms of frequency (percentage) to determine the number of participants in each subgroup of professors’ demographic characteristics and the corresponding percentages. In the second part, the reliability of the self-efficacy questionnaire was assessed using Cronbach’s alpha index, and the possibility of deleting an item was evaluated using the “Cronbach’s alpha if item deleted” criterion to enhance reliability. In the third part, the mean (standard deviation) of the participants’ self-efficacy score was reported by sex, marital status, and field of activity. The normality of the self-efficacy score distribution was checked using the same demographic variables. Based on the normalization test results, the hypothesis of equality of the average self-efficacy score across the subgroups of the demographic variables was tested with a t-test or an appropriate non-parametric method.

 Considering that the self-efficacy questionnaire has three dimensions, in the fourth part, the frequency of professors’ answers was reported in the form of a table or graph for all dimension items. In the fifth part, simple and multiple linear regression was fitted with the dependent variable of self-efficacy and the predictor variables of sex, work experience, field of activity and marital status, and statistically, a significant relationship between demographic variables and self-efficacy score was reported.

 The data were analyzed using the SPSS software, version 25. Descriptive statistics were calculated to describe the participant’s characteristics and the distribution of self-efficacy scores. Inferential statistical tests, including one-way ANOVA, Student t-test, and chi-square tests, were employed to test the differences in self-efficacy levels across different specialties and to examine the correlation between demographic variables and self-efficacy scores, respectively. We considered a *P* value of less than 0.05 to be statistically significant.

## Results

###  Demographic Characteristics of Participants

 A total of 203 faculty members participated in this study. The participants had an average (standard deviation) of 11.15 (8.89) years of work experience. The lowest experience was one year, and the most experienced teacher had 38 years of work experience. In Table 1, the participants were examined in terms of sex, field of activity, and marital status. According to the results, the majority of faculties are male and married. Regarding the field of activity, the field of medicine had the highest number of participants, and the field of radiology had the lowest participants.

**Table 1:** Descriptive statistics of participants’ demographic characteristics

**Variable**		**Frequency (Percent)**
Sex	Male	150 (74%)
	Female	52 (26%)
	Missing	1 (0.5%)
Marital status	Married	187 (92%)
	Single	15 (7%)
	Missing	1 (1%)
Field of activity	Medicine	134 (66%)
	Surgery	51 (25%)
	Radiology	6 (3%)
	Pathology	10 (5%)
	Missing	2 (1%)

###  Reliability of the Questionnaire

 The Cronbach’s alpha was equal to 0.94, which was considered excellent. Assessing the reliability table by removing the desired items showed that removing the specific item did not lead to a noticeable increase in reliability. Therefore, all items were used in the analysis of Self-Efficacy Scores.

 The average (SD) self-efficacy score of participants was 56.17 (14.62), with a minimum score of 22 and a maximum score of 110. Table 2 shows the mean (SD) self-efficacy scores of participants grouped by demographic characteristics.

**Table 2:** Average (SD) self-efficacy scores of participants by demographic characteristics

**Variable**	**Mean (SD)**	K-S*	***P***
Sex			
Male	57.07 (15.21)	0.200	0.106
Female	53.58 (12.60)	0.188	
Marital status			
Married	56.12 (14.56)	0.200	0.876
Single	56.73 (15.89)	0.200	
Field of activity			0.189
Medicine	54.86 (14.05)	0.200	
Surgery	59.00 (16.49)	0.200	
Radiology	64.17 (6.33)	0.097	
Pathology	55.50 (13.75)	0.200	

*K-S: Kolmogorov-Smirnov test. P values were estimated by an independent t-test and ANOVA.

 An independent samples t-test revealed no statistically significant difference in self-efficacy scores between both sexes (*P* = 0.106), married and singles (*P* = 0.876). One-factor variance analysis revealed no statistically significant difference between the mean scores of participants’ self-efficacy in different fields of activity (*P* = 0.189).

**Figure 1:**
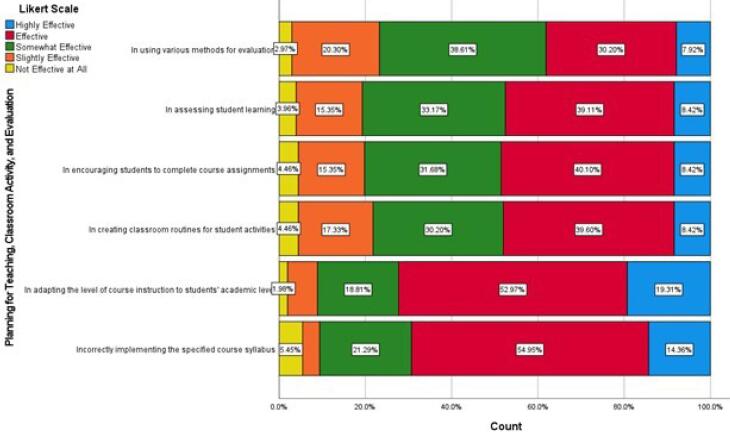
Mean self-efficacy scores of participants in different fields of activity, with 95% confidence intervals

Figure 1 depicts the mean self-efficacy scores of faculty members, according to field of activity, with a 95% confidence interval.

###  Frequency Analysis of Questionnaire Items Related to Self-Efficacy 

####  Dimension: Planning for Teaching, Classroom Activity, and Evaluation

**Figure 2:**
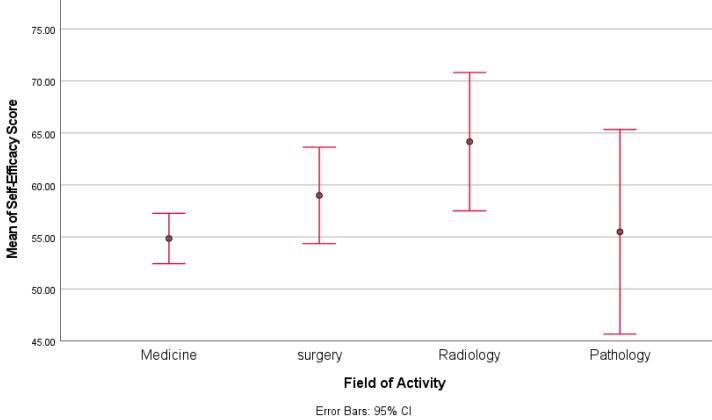
Frequency (percentage) of faculty opinions on items related to planning for teaching, classroom activities, and evaluation

Figure 2 presents the frequency of faculty opinions regarding the items included in this dimension of the questionnaire.

 More than two-thirds (69%) of the participants considered themselves to be effective and very effective in the question about correctly implementing the specified course syllabus. About 72%, evaluated themselves as effective and very effective in adapting the level of course instruction to students’ academic level. In classroom activities, approximately 5% of faculty members reported that they were not effective in creating classroom routines that enable students to perform activities. On the other hand, 48% of them evaluated themselves as influential and very influential. 5% of faculty members stated that they are not effective in encouraging students to complete their homework, but 48% considered themselves effective and very effective in this area. In evaluating students’ learning, 47% considered themselves influential or very influential, and approximately 19% considered themselves little or not at all influential. In the field of evaluation methods, 38% of the participants fell into the high range (influential and very influential), while approximately 23% were in the low range (little influence).

####  Dimension: Presentation and Classroom Management 

**Figure 3:**
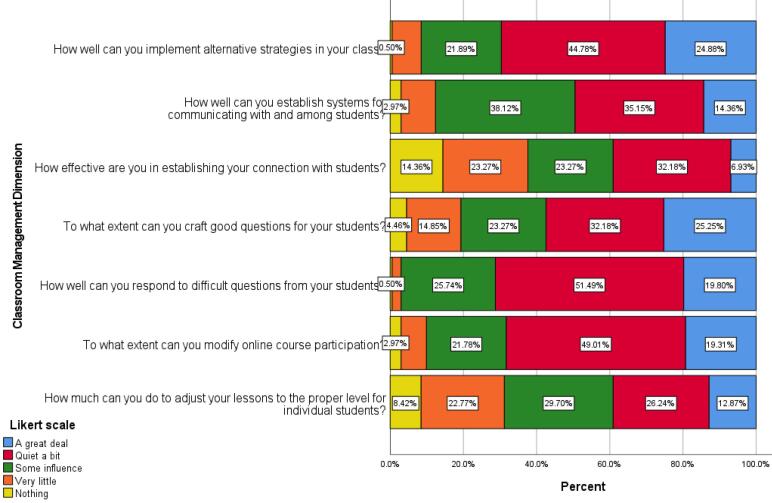
Faculty perceptions of self-efficacy in presentation and classroom management

 This dimension of the self-efficacy questionnaire aims to assess faculty members’ perceived ability in presenting and managing their classrooms. The bar chart visually represents faculty opinions on this aspect (Figure 3).

 The ability of faculties to provide different explanations with numerous examples, to propose suitable questions for students, and to answer challenging questions from students was well evaluated, as a large part of the answers have been entered in an influential and highly influential manner. Based on the chart, the effectiveness of faculty members in establishing communication between students and increasing student participation in online classes was weak. In other words, the professors in these items had mostly chosen the few or no influential answers.

####  Dimension: Teaching Outcomes

**Figure 4:**
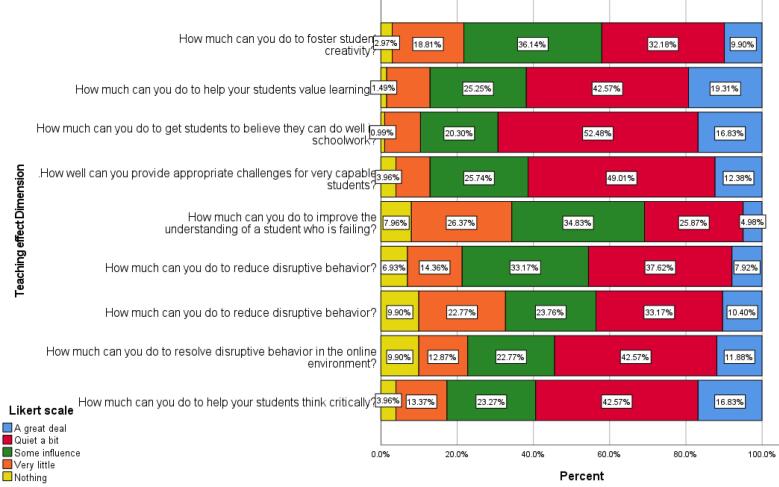
Faculty perceptions of self-efficacy in teaching outcomes

 This dimension examined the outcomes of teaching. This dimension included nine items, and the frequency of participants’ answers to each item is shown in Figure 4.

 As previously mentioned, high Influence or items with a predominantly blue and red color spectrum (representing “very highly influential” and “highly influential”) indicate greater faculty impact in that aspect. Based on this, faculty members rate themselves as highly or very highly influential in, making learning valuable from the students’ perspective (61%), instilling belief in students that they can succeed in their course (70%), creating appropriate challenges for capable students (61%), and promoting critical thinking among students (60%).

 According to the answers, about 34% of educators had evaluated themselves, particularly in improving the education of students who have failed in their courses, as low or not at all effective. In another issue related to dealing with students who disobey, about a third of the faculty members (33%) assessed themselves as unable to synchronize disobedient students with the class rules.

###  Analyzing the Relationship between Self-Efficacy Scores and Demographic Factors

 To examine the potential relations between self-efficacy scores and demographic variables, including sex, marital status, work experience, and field of expertise, scatter plots were presented.

**Figure 5:**
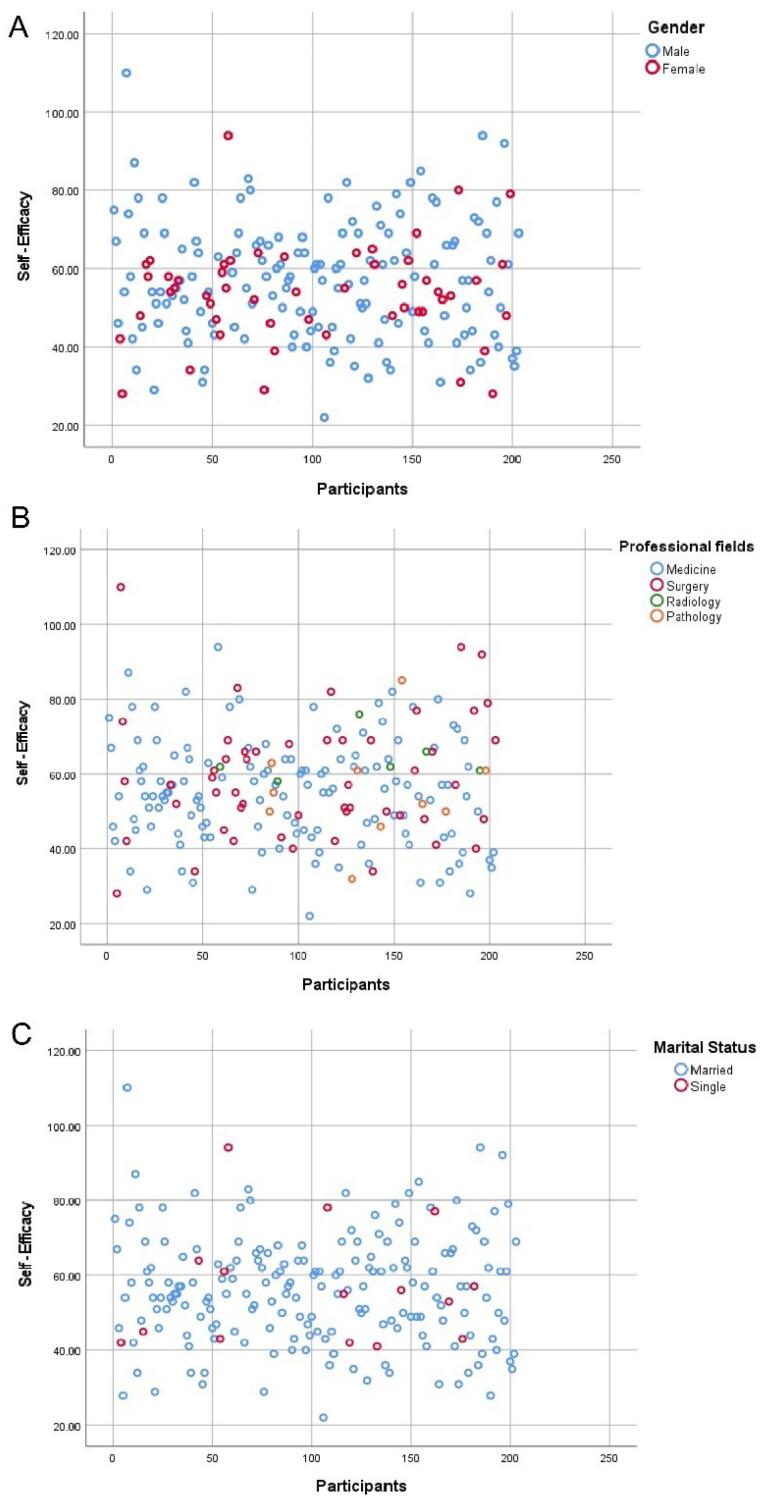
Scatter plot of self-efficacy scores by sex (A), professional fields (B), and marital status (C)

 Results revealed that the number of male participants with high self-efficacy scores was greater than that of females. Faculty members in the field of surgery also exhibited high self-efficacy scores. Conversely, faculty members in the field of medicine were more dispersed across the scatter plot, and the number of single faculty members was less than that of married faculty members (Figure 5).

**Figure 6:**
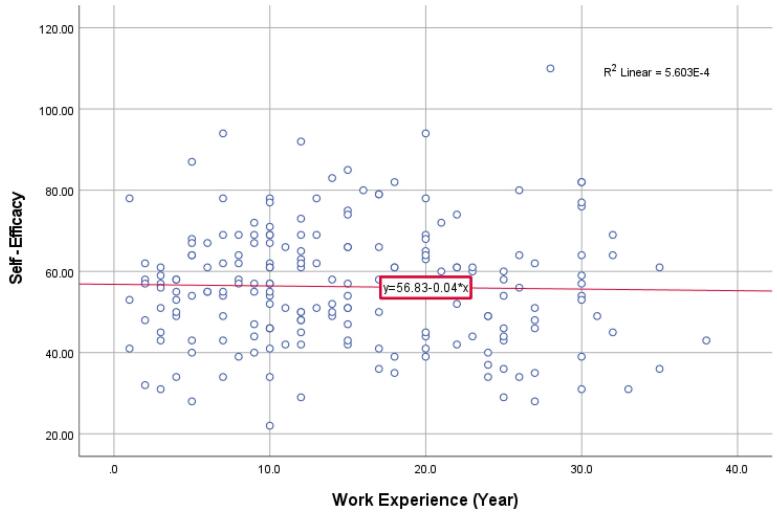
Scatter plot of self-efficacy scores and years of work experience

 According to the results, there is also no noticeable change in self-efficacy scores with an increase in faculty members’ experience. Consequently, the fitted regression line is a straight and horizontal (Figure 6).

 According to regression coefficients, there was no statistically significant relationship between the independent variables and the faculty members’ self-efficacy score (*P* > 0.05, Table 3).

**Table 3:** Table of regression coefficients and significance of independent variables in the model

**Independent variable**	**Regression coefficient**	**T-Statistic (Significance)**
Intercept	57.47	9.70 (*P* > 0.001)
Sex	-4.02	-1.64 (*P* = 0.103)
Work experience	-0.60	-0.50 (*P* = 0.620)
Field of activity	1.85	1.38 (*P* = 0.169)
Marital status	1.92	0.47 (*P* = 0.636)

## Discussion

 Our study evaluated the self-efficacy scores of clinical faculty members at SUMS in Iran after returning to in-person teaching following the COVID-19 pandemic. The results revealed that, although overall self-efficacy scores in online courses were high in the faculties, there were no significant differences between self-efficacy and sex, marital status, field of activity, or years of work experience.

 Our results indicate that SUMS faculty members reported high self-efficacy levels in online teaching, as measured by the TSES. This outcome aligns with preceding research suggesting that educators typically have high levels of self-efficacy, particularly in areas where they have received extensive training and practice. Recent research reveals that nursing faculty generally report high levels of self-efficacy in online teaching, as measured by various scales, including the Michigan Nurse Educator’s Sense of Efficacy for Online Teaching instrument.^11^ Like our study, this high self-efficacy is observed even during abrupt transitions to online formats, such as during the COVID-19 pandemic.^12^ Online professional development experiences have been shown to positively impact teacher self-efficacy.^13^ It is important to note that this study was conducted after a period of forced transition to online teaching due to the COVID-19 pandemic, suggesting that SUMS faculty members may have adapted well to the demands of online education. Despite these challenges, the transition to online education may drive a more progressive medical education agenda.^14^

 In this study, there was no statistically significant difference in the self-efficacy score of professors between men and women. Although this finding is consistent with the recent study’s observation, the observations of some other studies are not consistent. Research on sex differences in self-efficacy among academics shows mixed results. While some studies in the line of our study, found no significant differences between men and women in STEM (science, technology, engineering, and mathematics) majors’ self-efficacy^15^ others reported higher self-efficacy scores for male professors in course design, classroom management, and student feedback.^16^ In a study of university faculty, being female indirectly contributed to lower research self-efficacy, while being male was associated with higher service self-efficacy.^17^ These conflicting findings suggest that sex differences in self-efficacy scores may differ depending on the specific context, academic discipline, and cultural background.

 In this study, no significant difference was observed between married and unmarried faculty members in the field of self-efficacy in virtual teaching. In our country, a study conducted at another university examined the relationship between teaching self-efficacy and marital status. Batool et al^18^ found no significant differences in self-efficacy based on marital status, which is in line with the observation of our study. Other studies conducted in different cultural backgrounds also found no significant difference in self-efficacy in virtual teaching between married and unmarried faculty members.^19,20,21^

 Our study revealed no statistically significant differences in self-efficacy across fields of activity (medicine, surgery, pathology, and radiology) or years of experience. Research on self-efficacy in medical education reveals varied findings across specialties and training levels.^22^ Artino et al created a validated survey to measure medical students’ self-efficacy across core competencies, demonstrating significant increases from year 1 to year 4.^23^ Salles believed that the lack of statistically significant differences in self-efficacy across medical fields might be due to a limited sample size.^24^

 The findings of the present study were the lack of a statistically significant relationship between self-efficacy and factors such as sex, marital status, work experience, and field of specialty. Relevant studies suggest that while certain demographic factors may influence self-efficacy in virtual teaching, the impact varies across studies and contexts, emphasizing the need for further research to understand the complex interplay of factors affecting faculty readiness for online instruction.

 There are some limitations in our study. The lack of ability to prove a cause-and-effect relationship due to the cross-sectional design, the use of a single University, which may affect the generalizability of the findings, and self-reported data from faculty members, which may be subject to social desirability bias, are the most common problems that should be considered when applying the results.

## Conclusion

 This study provides valuable insights into the self-efficacy of SUMS faculty members in online education following the COVID-19 pandemic. While the study recommends that overall self-efficacy levels are moderately high, there is a need for continued investment in faculty development and support to establish effective online teaching practices and address the evolving needs of a changing educational landscape.
